# Vulvar and anogenital ulcerations induced by amivantamab: A case series

**DOI:** 10.1016/j.jdcr.2026.01.015

**Published:** 2026-01-20

**Authors:** Sukul Mittal, Elissa J. Goorman, Joshua Prenner, Samantha Guhan, Lida Zheng, Jennifer N. Choi, Cuong V. Nguyen

**Affiliations:** Department of Dermatology, Northwestern University Feinberg School of Medicine, Chicago, Illinois

**Keywords:** amivantamab, anogenital, bispecific therapy, cutaneous toxicities, epidermal growth factor receptor, mesenchymal–epithelial transition, ulcerations, vulvovaginal

Amivantamab is a bispecific monoclonal antibody targeting epidermal growth factor receptor (EGFR) and mesenchymal–epithelial transition factor, approved for the treatment of advanced non–small cell lung cancer with EGFR exon 20 insertion mutations.^1^ The cutaneous toxicities associated with EGFR inhibitors are well-documented and vary, including pruritus, papulopustular eruptions, paronychia, xerosis, and mucositis.^2^ Scalp involvement has emerged as a notable toxicity of amivantamab, manifesting as scalp folliculitis and erosive pustular dermatosis.^2^ Further, amivantamab has been implicated in erosive and ulcerative cutaneous reactions, including a recent report of penile and inguinal ulcerations.^3^ Involvement of the vulvar mucosa has not been previously documented. Herein, we report 5 cases of female patients who experienced painful anogenital ulcerations during treatment with amivantamab for stage IV lung carcinoma, expanding the reported spectrum of cutaneous toxicities associated with amivantamab.

The patients ranged in age from 48 to 72 years old. In patients 2 to 5, anogenital eruptions developed within 4 to 8 weeks of treatment with amivantamab. However, patient 1 did not develop symptoms until 9 months after initiation of amivantamab. All 5 patients presented with erythematous papules and shallow, circular, punched-out ulcerations involving the labia majora, labia minora, mons pubis, and/or perianal skin (Fig 1, *A-D*). Four patients reported associated symptoms of genital pruritus and bleeding. Patient 2 presented with additional punched-out ulcerations of the inframammary folds, hemorrhagic-crusted papules and pustules on the face and scalp, and pyogenic granulomas on the nailfolds. Histopathology from a breast lesion in this patient revealed an ulcer with an interstitial predominantly neutrophilic infiltrate with focal pustule formation, consistent with an EGFR-inhibitor–associated pustular eruption. In another patient from our cohort, biopsies were performed of the perianal region and of the right buttock. Histopathology demonstrated ulceration with granulation tissue and no viral cytopathic changes, consistent with a nonspecific ulcer pattern. Polymerase chain reaction testing for herpes and varicella-zoster viruses was negative in all patients, and lesion appearance with treatment timing favored a drug-related process.

**Fig 1:**
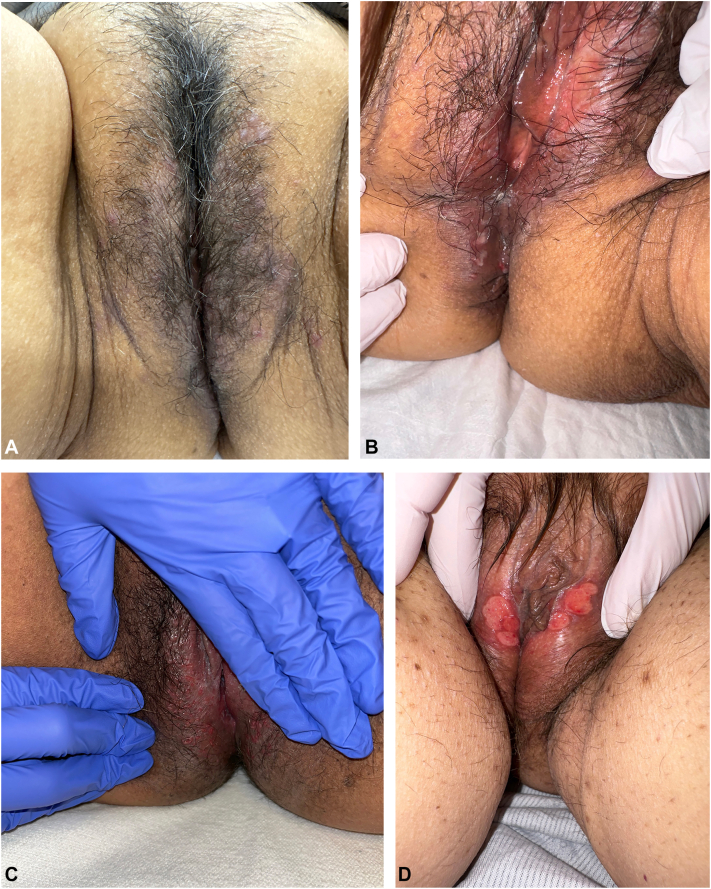
Representative photos of anogenital ulcers. **A, B,** At initial presentation, the patient presented with 2 to 3 mm erythematous, folliculocentric papules on the mons pubis with shallow ulcerations and lateral fissures on the labia majora and minora. **C,** A second patient with multiple punched-out ulcers involving the labia majora and minora. **D,** A third patient with punched-out ulcers involving the labia majora.

Because of the severity of presentation in 4 patients, amivantamab was held. Four patients were found to be zinc deficient (range of serum levels: 36-55 mcg/dL, normal 60-120 mcg/dL), with concurrent iron deficiency (range of iron saturation: 4%-14%, normal 20%-50%). All patients were treated with both topical and oral antibiotics, barrier care, and oral zinc supplementation. Following these interventions, patients 1 and 4 had near-complete resolution after 14 days; patients 2 and 3 had a partial response seen after 14 days. After 1 to 2 months off treatment, therapy was successfully reintroduced in 3 patients for whom treatment has been held, with 1 patient resuming at a 30% dose reduction. Zinc levels improved in 4 patients (52-76 mcg/dL) after 3 to 5 months of zinc supplementation. Baseline zinc levels were not available for the fifth patient.

The pathophysiology of amivantamab-induced anogenital ulcerations is not known. Other EGFR inhibitor–induced toxicities such as xerosis and dermatitis are thought to arise from keratinocyte growth arrest, premature differentiation, epidermal barrier disruption, inflammation, and altered skin immunity.^4^ Potentially compounding the effects of these cutaneous adverse events is the cooccurrence of zinc deficiency. Zinc supports epithelial homeostasis, skin inflammation, and wound repair.^4^ Its deficiency has been implicated in EGFRi-induced cutaneous toxicities.^4^^,^^5^ In murine models, Lu et al^4^ found that treatment with erlotinib (15 mg/kg/d), an EGFR-tyrosine kinase inhibitor, led to progressive periorificial dermatitis. Zinc levels in treated rats significantly declined by day 14 (95.7 ± 24.5 μg/dL) compared to controls (146.3 ± 18.5 μg/dL, *P* < .05), establishing a mechanistic link between EGFR-tyrosine kinase inhibitors and zinc deficiency.^4^ These findings were extended to humans; zinc supplementation in 55 patients on EGFR-tyrosine kinase inhibitors led to a substantial increase in serum zinc levels and statistically significant improvement in Common Terminology Criteria for Adverse Events grading of mucocutaneous lesions within 14 to 48 days.^4^ Further, Tohyama et al^5^ found that among 21 patients receiving zinc supplementation for over 2 months, EGFR inhibitor–induced xerotic dermatitis markedly improved in 16 patients alongside increased serum zinc levels. Although vulvar ulcerations have not been directly linked to zinc deficiency, their persistence in our cohort supports the possible role of micronutrient evaluation and supplementation as supportive care.

Our cases of diffuse anogenital ulcers secondary to amivantamab expand our understanding of this therapy’s mucocutaneous toxicities. Arana et al^2^ reported that amivantamab dose modification or therapy cessation due to cutaneous toxicities was required in 19.2% (14/73) of patients, most commonly for acneiform rash (57.1%), paronychia (35.7%), and erosive pustular dermatosis (28.5%). Our series illustrates that anogenital ulcers due to amivantamab may similarly require treatment interruption or adjustment. Given that decisions to modify cancer-directed therapy are critical considerations, awareness of these mucocutaneous toxicities, alongside other reported dermatologic toxicities, is essential. Additionally, as zinc deficiency was identified in 4 patients, its role in relation to EGFR/mesenchymal–epithelial transition factor inhibition, wound healing, and as a therapy warrants further investigation.

## Conflict of interest

None disclosed.
